# Minority stress, depression, and cigarette smoking among Chinese gay versus bisexual men: a two-group structural equation model analyses

**DOI:** 10.1186/s12889-021-10888-5

**Published:** 2021-07-09

**Authors:** Jingjing Li, Danqin Huang, Michael Windle, Cam Escoffery, Wei Wang, Xiaoyan Li, Kevin Tao, Regine Haardörfer, Shiyue Li, Carla J. Berg, Hong Yan

**Affiliations:** 1 Department of Behavioral Sciences and Health Education, Rollins School of Public Health, Emory University, Emory, USA; 2 Department of Preventive Medicine, School of Health Sciences, Wuhan University, 185 Donghu Road, Wuhan, 430071 Hubei People’s Republic of China; 3 School of Public Health, Xuzhou Medical University, Xuzhou, 221004 Jiangsu People’s Republic of China; 4 The Wallace H. Coulter Department of Biomedical Engineering, Georgia Institute of Technology, Atlanta, USA; 5 Department of Preventive and Community Health, Milken School of Public Health, George Washington University, Washington, DC, USA; 6 George Washington Cancer Center, George Washington University, Washington, DC, USA

**Keywords:** Minority stress, Depression, Cigarette, Smoking, Gay men, Bisexual men, China

## Abstract

**Background:**

Literature in the West suggested that bisexual men have a higher smoking rate compared to gay men. Data on patterns of smoking among gay and bisexual men are limited in Eastern Asian countries like China. This study examined the cigarette smoking prevalence for gay versus bisexual men in China and their unique minority stress - smoking pathways.

**Methods:**

Between September 2017 and November 2018, we surveyed a convenience sample of 538 gay men and 138 bisexual men recruited from local sexual minority organizations in four metropolitan cities in China (i.e., Beijing, Wuhan, Nanchang, and Changsha). Measures included sexual orientation, sociodemographics, theory-based minority stressors, depressive symptoms, and past 30-day cigarette smoking. Two-group (gay men vs. bisexual men) structural equation modeling (SEM) was used to test possible distinct mechanisms between theory-based stressors, depressive symptoms, and cigarette smoking among gay men and bisexual men, respectively.

**Results:**

The mean age of participants was 26.51 (SD = 8.41) years old and 76.3% of them had at least a college degree. Bisexual men reported a higher rate of cigarette smoking compared to gay men (39.9% vs. 27.3%). Two-group SEM indicated that the pathways for cigarette smoking were not different between gay and bisexual men. Higher rejection anticipation was associated with greater depressive symptoms (standardized β = 0.32, *p* < .001), and depressive symptoms were not associated with cigarette smoking.

**Conclusions:**

Minority stress, specifically rejection anticipation, may be critical considerations in addressing depressive symptoms, but not smoking, among both gay and bisexual men in China.

**Supplementary Information:**

The online version contains supplementary material available at 10.1186/s12889-021-10888-5.

## Background

China has one of the highest burden of smoking in the world [1]. Each year, approximately 1,000,000 people in China die prematurely from smoking-related diseases [2, 3]. Men represent a disproportionate number of China’s smokers. According to the 2015 China Adult Tobacco Survey, on average, 51.4% Chinese adult (aged 15 and older) men were current cigarette smokers, while 2.7% of adult women smoke [4]. While several Chinese metropolitan cities have passed increasingly progressive municipal-level tobacco control regulations, the prevalence of males who currently smoke cigarettes in these localities is still high, ranging from 32.7 to 44.5% [5].

Data from Canada, the U.S. and Australia has extensively studied tobacco use among sexual minority populations – or lesbians, gays, and bisexuals (LGB). This literature has largely indicated high rates of cigarette use among sexual minorities compared to the general populations [6–12]. While the literature regarding smoking among sexual minorities in Eastern Asian countries like China is limited, some studies have similarly shown increased smoking prevalence in this population. A 2015 study conducted in Shanghai, China found that gay and bisexual boys were more likely to smoke cigarette compared to heterosexual boys (OR = 2.2), while lesbian and bisexual girls reported less any prior cigarette smoking compared to heterosexual girls (OR = 0.7) [13]. Another study focusing on Chinese adult gay and bisexual men indicated a cigarette smoking prevalence of 66%, [14] approximately 15% higher than the national average and 22–33% higher than the city-level prevalence [5]. Gay and bisexual men represent a large subgroup in China. It was estimated that the total population of Chinese gay and bisexual men is around 18 (10.2–25.4) million [15]. However, limited research has focused on the heightened smoking rates among this large population of gay and bisexual men in China.

Minority Stress Theory (Meyer, 1995; 2003) is a framework for examining and understanding mechanism of high-risk behaviors in sexual minorities. Specifically, Minority Stress Theory views hostile social conditions as causes of stress for sexual minority groups [16, 17]. Meyer (1995; 2003) suggested that minority stress can take the forms of distal minority stress, or external processes and experiences faced by sexual minorities, including discrimination experiences; and proximal stress, or internal processes and experiences such as outness, rejection anticipation, identity concealment, and internalized homophobia [16, 17]. These stressors can exacerbate mental health problems and consequently lead to increased health risk behaviors [18, 19]. Empirically, numerous studies have shown that these minority stressors are positively associated with psychological distress and health risk behaviors such as tobacco use [9, 20–24]. For example, Burgess et al. (2008) pointed out that distal stressors such as discrimination experience was associated with greater likelihood of depression, anxiety, greater perceived needs for mental healthcare, and more frequent use of mental health services [25]. Others focusing on proximal stressors like internalized homophobia or outness showed that these stressors were signification predictors of mental health problems [26–29]. However, few studies have focused on minor frustrations and annoyance such as everyday discrimination that could produce long-lasting feelings of rejection [30], and one main reason for sexual minorities to use substance including cigarettes was the feeling of rejection [31]. As multiple stressors increasingly challenge an individual’s coping capabilities, substance use such as smoking may serve as a coping strategy for sexual minorities who experience “minority stress” [10, 16]. Conversely, positive coping such as resilience and coping resources such as social support were found to buffer the negative effects of minority stress on adverse health outcomes [32–36].

Contextual factors such as culture can be specific manifestation of Minority Stress Theory. Researchers suggested that Chinese culture is more collectivism that weights interpersonal bonds over individual themselves and emphasized a greater responsibility to the collective goods [37]. Lai (2010) and others believe part of the Chinese culture is influenced by Confucianism, a Chinese ancient philosophy that strongly emphasizes duty, obedience, filial piety, and certain ethical and moral values [38, 39]. In research on Chinese contemporary sex norms, Li (2006) outlined that, cultivated in a collective culture, Chinese society sees the most commonly conducted behaviors as meeting the moral standards while less frequent behaviors would be viewed as ‘abnormal’ and ‘unethical’ [40]. Same-sex marriage is still illegal in China [41]. As a result, a sexual minority who does not act like his or her peers by not getting married at a certain age will become a concern and shame to the whole family for being deviant from the majority, and for failing to continue the family line [42]. Wang et al. (2015) found that gay/bisexual men in a qualitative study described constant pressure from family, social, and workplace to have a female partner, and they also stated the difficulties to establish stable same-sex relationships [43]. In 2020, researchers qualitatively examined minority stress among 24 Chinese sexual minority men and found that all participants suggested that Chinese culture (and cultural norm) functioned as source and context of minority stress [44]. Earlier study on Asian sexual minorities found same-sex behaviors would only be tolerant when they fulfill family duties including eventual heterosexual marriage [45]. In China, if men get older but are not married, family members, coworkers, peers and community acquaintances would start inquiring about their reasons for not getting married and even consider them as abnormal [46].

To date, the majority of research examining tobacco use through the lens of Minority Stress Theory has been done in North America [6, 47, 48]. In Southeast Asian countries, Minority Stress Theory was also used to inform study examining depression, suicide, and addictive behaviors among sexual minorities in Indonesia, Malaysia, Myanmar, Thailand, and Vietnam [49]. In Eastern Asian countries like China, the Minority Stress Theory has been almost exclusively applied to predicting physiological outcomes [50–53]. Although few Chinese studies have explicitly examined the mechanism between minority stress and cigarette use [13, 14, 54], qualitative research has suggested that Chinese sexual minority men may use substances to relieve the stress of hostile social stigma, as well as familial and cultural pressures [55]. Therefore, it is important to empirically examine specific pathways through which minority stressors might influence cigarette use among Chinese sexual minorities, particularly gay and bisexual men.

Particularly relevant to the current study, not all sexual minorities experience the same stressors or related health risks. In North America, evidence across studies indicates significant inter-group difference across sexual minority subgroups that bisexuals are at increased risk for mental health problems and substance use compared to monosexuals (i.e., gay/lesbians and heterosexuals) [47, 56]. Such intergroup differences between bisexuals and monosexuals can be explained by the unique minority stressors that bisexuals experience. For instance, bisexually-identified individuals might often be assumed to be gay/lesbian or heterosexual depending on the sex of their partners [56]. Thus, bisexual people might anticipate others to dismiss their bisexual identity; this anticipation may contribute to depressive symptoms and mental distress [56, 57]. Other research by Sweet and Welles (2012) indicates that bisexual men may experience significantly more adverse childhood experiences compared to gay men [58]. However, in contrast to the growing body of literature examining the heterogeneity of sexual minority stressors and subsequent psychological and health behavior risks across sexual minority subgroups in North America, very little is known about this topic in China. One 2015 paper by Lian et al. in China studied smoking among sexual minority youth and actually found the reverse pattern, that male bisexual and heterosexual youth shared similar smoking patterns while gay youth reported higher smoking rates compared to heterosexual counterparts [13]. Lian and colleagues believed that bisexual youth in China might be less involved with LGB activities with higher risk for smoking exposure (e.g., pride events), compared to gay youth; or, bisexual youth in China might be less likely to cope minority stress with smoking [13].

To date, it is unclear whether significant heterogeneity exist between Chinese gay and bisexual men regarding their minority stress experiences and related health risks. This study aimed to contribute to the literature by examining the prevalence of cigarette smoking in a sample of Chinese gay and bisexual men and their distinct minority stress - smoking pathways, using the two-group (gay vs. bisexual men) structural equation modeling (SEM). This research is critical, as the exploration of minority stressors and pathways among subgroups of sexual minority men may help advance our understanding of cigarette use behavior among high-risk populations in China and develop effective tobacco cessation interventions.

## Methods

### Study population and data collection

The authors obtained data from a survey study focusing on health risk behaviors of Chinese sexual minority men conducted by School of Health Sciences, Wuhan University, China. Details of the survey methodology have been published elsewhere [58]. Briefly, between September 2017 and January 2018, we collected baseline survey data of 755 self-identified gay and bisexual men using venue-based sampling from college campus-based sexual minority-serving organizations in four cities, namely Beijing, Wuhan, Nanchang, and Changsha, as these cities have high concentration of LGBT populations. We collected these data using a questionnaire developed by our research team for the purposes of this study (see Additional file 1***).*** These data are among the most recent data available regarding tobacco use among sexual minority men in China. This convenient sample was mainly comprised of urban, well-educated, and high-income gay/bisexual men, and might not be representative to all sexual minority men. After screening for eligibility (self-identified as gay/bisexual men and aged 16 years or older), participants were given a brief explanation of the survey’s purpose. Informed consent was obtained from every participant. Each participant received a one-time compensation of 50 Chinese RMB (approximately $8 US dollars) for their time. For this study, we excluded 79 (10.5%) participants who reported “heterosexual/unsure/other” sexual orientation. Participants involved in tobacco cessation programs, including HIV-positive individuals who were linked to HIV care and provided with behavioral intervention (i.e., tobacco cessation services) [59], were excluded from this study. The analytical sample size for this study was 676. This study was approved by the Institutional Review Board (IRB) of Wuhan University Medical School, China.

### Measures

#### Primary outcome: cigarette smoking

Participants were asked, “During the past 30 days, on how many days did you smoke cigarettes? Little cigars or cigarillos? Traditional pipe? Chewing tobacco? E-cigarettes? Hookah?” Given that fewer than 10 participants reported on alternative tobacco use (i.e., little cigars or cigarillos, traditional pipe, chewing tobacco, e-cigarette, and hookah), this study only focused on the past 30-day cigarette smoking outcome, dichotomized as “no” versus “any smoking.”

#### Primary grouping variable: sexual orientation

Sexual orientation was assessed by asking, “Is your sexual orientation: heterosexual; gay or homosexual; bisexual; or unsure?” All responses were coded dichotomously (0 = gay and 1 = bisexual).

#### Sociodemographic characteristics

Participants were asked to provide sociodemographic information, including age, education (high school/below vs. college/above), place of origin (urban vs. rural), employment (dummy coded into student, employed, and unemployed), marital status (unmarried/divorced vs. married), monthly income (≤ 3000 RMB [73 USD] vs. > 3000 RMB, note that the minimum monthly wage ranges between 1580 and 2000 RMB in 4 sampled cities [60]), and nature of their health insurance (yes vs. no/unsure).

#### Depressive symptoms

**Fig. 1:**
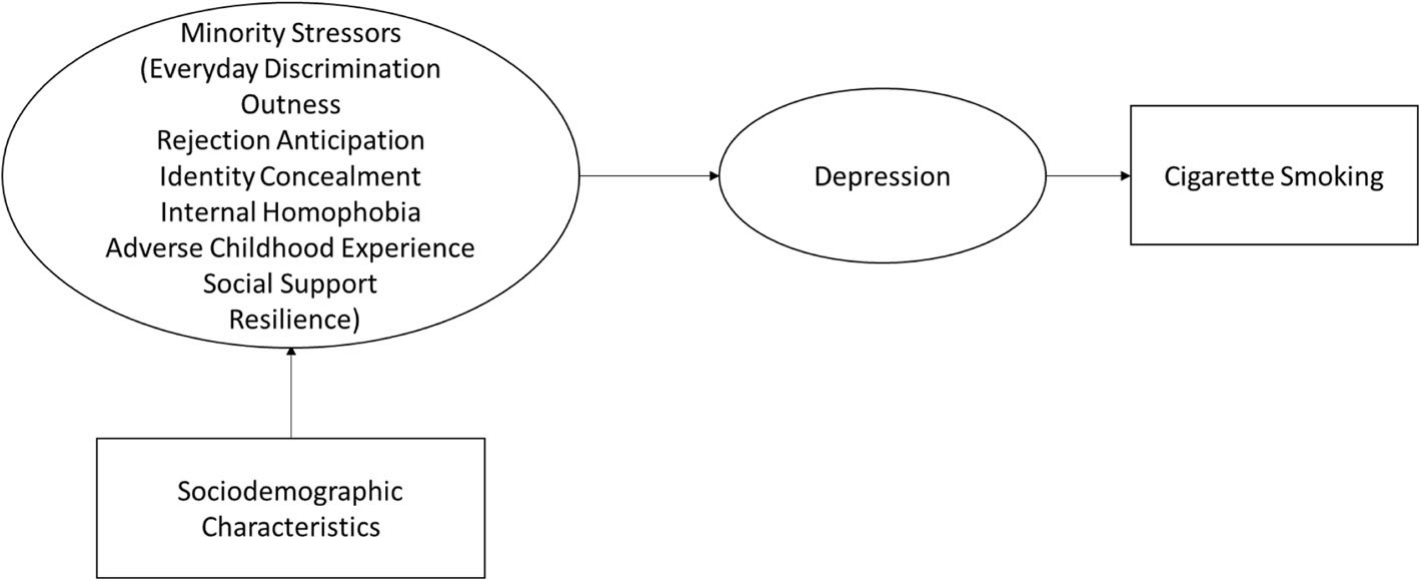
Hypothesized conceptual model of minority stressor to cigarette smoking among gay and bisexual men in China

Depressive symptoms were hypothesized as the pathway between minority stressors and cigarette smoking (Fig. 1) and were assessed with the Center for Epidemiological Studies Depression (CES-D) scale [61]. The CES-D is a well-established and widely used [62] 20-item scale designed to measure depressive symptoms experienced by the individual within the past week, and its Chinese version has been validated [63, 64]. Items were answered on a 4-point scale ranging from 0 = *less than a day or never* to 3 = *5–7 days*. The Cronbach’s alpha was 0.89.

#### Minority stressors

Informed by Minority Stress Theory [16, 17], we measured distal minority stressors (everyday discrimination), proximal minority stressors (outness, rejection anticipation, identity concealment, and internalized homophobia), general stressors (adverse childhood experiences), and stress-moderating factors (social support and resilience).

*Everyday discrimination* was assessed using the Everyday Discrimination Scale [65]. This scale asks about the frequency of 9 types of hassles and prejudice events that sexual minority people may encounter. This 9-item scale is rated on a 6-point Likert scale with response options of 1 = *happens daily* to 6 = *never happened*. All responses were reverse coded and averaged to create a mean score, with higher scores indicating severer everyday discrimination. The Cronbach’s alpha coefficient was 0.94 for this scale.

*Outness* was assessed by asking respondents “Have you ever ‘come out’ to anyone?” All responses were coded dichotomously (0 = *no* and 1 = *yes*).

*Rejection anticipation* was assessed with a scale which was originally used to assess stigma of mental illness [66]. Later, this scale was modified and adapted by Forst et al. (2015) for assessing sexual minority’s state of hypervigilance and worry about being rejected [21]. This 6-item scale is rated on a 5-point Likert scale, ranging from 1 = *applies very strongly* to 5 = *does not apply at all*. All responses were reverse coded and averaged to create a mean score, with higher scores indicating higher expectation of rejection. The Cronbach’s alpha of this scale in this study was 0.88.

*Identity concealment* was assessed using a subscale on nondisclosure developed and validated by Testa et al.(2015) [67]. This 6-item scale asks about the intentions and behaviors of sexual minority individuals to avoid disclose their sexual minority identities. This scale is rated on a 5-point Likert scale, ranging from 1 = *applies very strongly* to 5 = *does not apply at all*. All responses were reverse coded and averaged to create a mean score, with higher scores indicating greater identity concealment. The Cronbach’s alpha was 0.91.

*Internalized homophobia* was assessed using the Internalized Homophobia Scale which was originally developed by Martin and Dean (1987) [68] and further modified and validated by Forst et al. (2009; 2015) [21, 69]. This scale asks about the negative attitudes sexual minorities hold against their own sexual identities. This 8-item scale is rated on a 4-point Likert scale, ranging from 1 = *never* to 4 = *always*. Responses were averaged to create a mean score, with higher scores indicating greater internal homophobia. The Cronbach’s alpha was 0.89.

*Adverse childhood experiences (ACEs)* was assessed using a 10-item ACEs index developed by the U.S. Centers for Disease Control and Prevention [70, 71]. This index asks about the physical and mental abuse and traumatic experiences of participants prior to 18 years old. Participants answered 0 = *no* or 1 = *yes* to each item. Total score of all responses was summed; higher scores indicate more ACEs. The Cronbach’s alpha was 0.63.

*Social support* was assessed with the Multidimensional Scale of Perceived Social Support, [72] which measures perceived support from family, friends, and significant others. This 12-item scale is rated on a 7-point Likert scale, ranging from 1 = *very strongly disagree* to 7 = *very strongly agree*. The total score of this scale was calculated for each participant, with higher scores indicating more social support. The Cronbach’s alpha was 0.94.

*Resilience* was assessed with the 10-item Connor-Davidson Resilience scale [73, 74]. This scale is a measure of stress coping capabilities. It is rated on a 5-point Likert scale ranging from 1 = *not true at all true* to 5 = *true nearly all of the time*. The responses are summed to derive a total score, with higher scores indicating more resilience. The Cronbach’s alpha was 0.95.

Please see Additional file 1 for more details about the survey questionnaire used in this study.

### Statistical analysis

Univariate analyses were conducted to examine the distribution of each variable. ANOVA and chi-square tests were conducted to assess the bivariate relationships of cigarette smoking outcome and sexual orientation to socio-demographic and psychosocial variables. We also examined the multi-collinearity between all variables. In consideration of study power, we excluded selected sociodemographic variables (i.e., education, marriage, monthly income, and health insurance) from the modeling analyses, as these variables either were not associated with the outcome or showed considerable collinearity (data not shown).

Next, we conducted sequential logistic regressions to identify significant associations between predictors and cigarette use to inform the approach to the structural equation models (SEMs). Specifically, we assessed the effects of sexual orientation on cigarette use in following models: (1) only included sexual orientation as predictor; (2) added other sociodemographic predictors (i.e., age, place of origin, and employment); (3) added psychosocial risk factors (i.e., everyday discrimination, outness, rejection anticipation, identity concealment, internalized homophobia, and ACEs), and (4) added psychosocial protective factors (i.e., social support and resilience). Collinearity between sexual orientation and all psychosocial factors was examined using Condition Index (CI) derived from the inverse of the Information Matrix [75] as well as the Variable Inflation Factors. No sign of collinearity was found (data not shown).

**Fig. 2:**
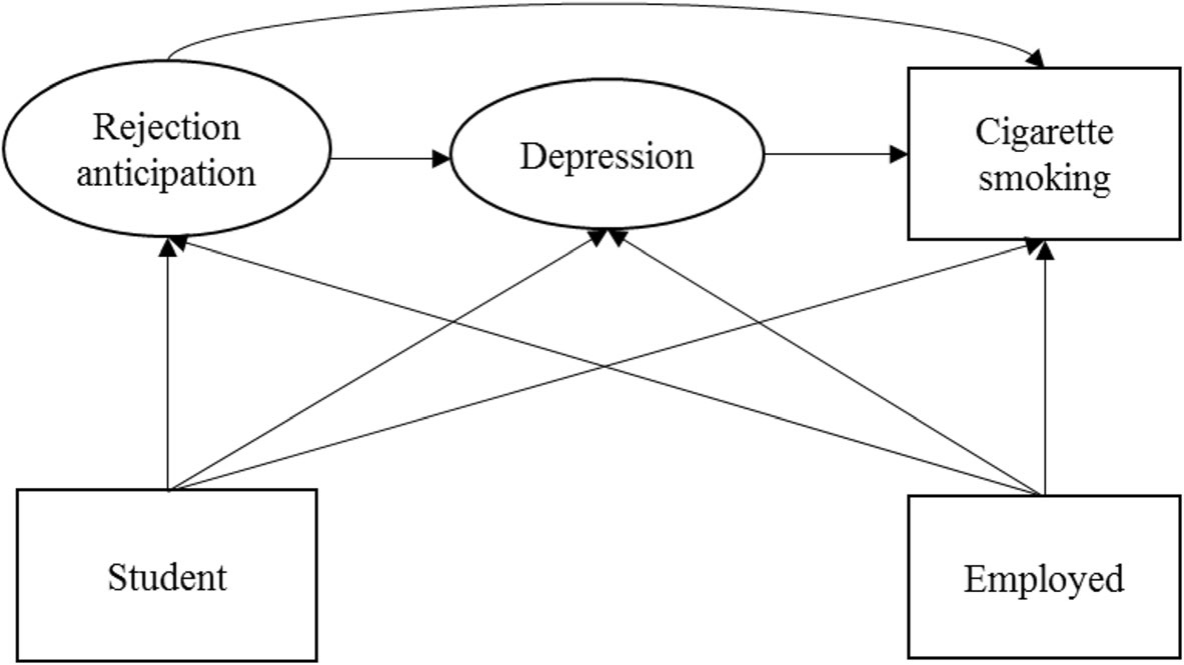
Specified two-group structural equation model of rejection anticipation to cigarette smoking for both gay and bisexual men in China

Two-group SEM is a way to compare the results from two groups simultaneously. The goal of two-group SEM is to examine whether the relationship of interest among predictor and response variables vary by group. The benefit of two-group SEM is that it could identify which paths change based on the group membership and which do not. Thus, we applied two-group SEM as our main analytical approach [76]. For constructing the two-group SEM, we specified the SEM model in Fig. 2 based on Minority Stress Theory, preliminary analyses, and correlation matrix results. SEM is a process that allows for testing one or more theories that are hypothesized a prior to explain the characteristics of measured variables [59]. SEM can be used for model confirmatory purpose, testing alternative models, or model generation [60]. Two-group SEM can be used to examine inter-group differences across sexual minority subgroups with increased rigor. The advantage of two-group SEM is that it allows the comparison of the extent of associations based on path coefficients [61] and uses model fit indices to determine which tested paths best fit the data. This method could help examine whether the underlying pathway are significantly different among sexual minority subgroups. Given the goals of this study, two-group SEM is a particularly useful tool for examining how different pathways might vary across sexual orientation. A two-phase modeling approach was used for the two-group SEM. First, we examined measurement invariance (i.e., item-scale relationships) between gay and bisexual participants using confirmatory factor analysis (CFA). Second, we examined structural invariance (i.e., hypothesized relationships among variables) between gay and bisexual participants. Chi-square differences between these two models were examined and indicated non-significant results and thus no group differences in the measurement models [77].

Then, we examined the model fit and path-coefficients of the final two-group structural SEM. The model fit indices included: chi-square test, standardized root mean square residual (SRMR), comparative fit index (CFI), Tucker–Lewis fit index (TLI), the root mean-square error of approximation (RMSEA), and weighted root mean square residual (WRMR). The indicators of goodness of fit were: χ2 *p* < 0.05; SRMR > 0.08; CFI > 0.90; TLI > 0.90; RMSEA < 0.05; and WRMR < 1 [78]. The bootstrapping method was used to test 95% Confidence Interval (95% CI). Standardized regression (β) coefficients, the standard errors, and *p*-values for β were reported in the final model.

Data were double-entered and cleaned using EpiData 3.1 (The EpiData Association, Odense, Denmark) software. Descriptive analysis and sequential regressions were conducted using SAS 9.4 (SAS Institute Inc.: Cary, NC, USA). Two-group SEM was conducted using Mplus 8.0 (Muthén & Muthén: Los Angeles, CA, USA).

## Results

### Descriptive statistics

Table 1 shows that, among the full sample, the proportion of cigarette use was 29.9%. The mean age of the participants was 26.51 (SD = 8.41) years old, 76.3% reported holding college degree or above, 31.5% were current students, and 58.6% were employed. Bisexual men reported a higher rate of cigarette smoking compared to gay men (39.9% vs. 27.3%). Rejection anticipation was significantly associated with cigarette smoking. Because marriage and health insurance were not associated with cigarette smoking, these variables were excluded from subsequent modeling analyses. Because education and monthly income correlated with employment, we included employment but excluded education and monthly income in the subsequent modeling.

**Table 1:** Descriptive characteristics and bivariate analyses examining differences between past 30-day cigarette smoking (*N* = 202) vs. non-smoking (*N* = 474) and between gay vs. bisexual orientation in Chinese gay and bisexual men (*N* = 676)

Variables	Total	Smoking Status		***p***	Sexual orientation		
	Sample M (SD) or ***N*** (%) ***N*** = 676	Smoking M (SD) or ***N*** (%) ***N*** = 202	Nonsmoking M (SD) or ***N*** (%) ***N*** = 474		Gay M (SD) or ***N*** (%) ***N*** = 538	Bisexual M (SD) or ***N*** (%) ***N*** = 138	***p***
***Sexual Orientation (%)***							
Gay	538 (79.6)	147 (72.8)	391 (82.5)	**.004**	**/**	**/**	**/**
Bisexual	138 (20.4)	55 (27.2)	83 (17.5)				
***Sociodemographics***							
Age (SD)	26.51 (8.41)	27.33 (9.35)	26.16 (7.95)	.102	25.66 (7.40)	29.84 (10.95)	**<.001**
Education (%)							
High school or below	160 (23.7%)	72 (35.6)	88 (18.6)	**<.001**	112 (20.8)	48 (34.8)	**<.001**
College or above	516 (76.3%)	130 (64.4)	386 (81.4)		426 (79.2)	90 (65.2)	
Place of Origin (%)							
Urban	412 (61.0)	118 (58.4)	294 (62.0)	.379	336 (62.4)	76 (55.1)	.113
Rural	264 (39.0)	84 (41.6)	180 (38.0)		202 (37.6)	62 (44.9)	
Employment (%)							
Student	213 (31.5)	40 (19.8)	173 (36.5)	**<.001**	184 (34.2)	29 (21.0)	**.009**
Employed	396 (58.6)	131 (64.9)	265 (55.9)		305 (56.7)	91 (65.9)	
Unemployed	67 (9.9)	31 (15.3)	36 (7.6)		49 (9.1)	18 (13.0)	
Marital Status (%)							
Unmarried/divorced	602 (89.1)	181 (89.6)	421 (88.8)	.764	500 (92.9)	102 (73.9)	**<.001**
Married	74 (10.9)	21 (10.4)	53 (11.2)		38 (7.1)	36 (26.1)	
Monthly Income in RMB (%)							
≤ 3000 (73 USD)	352 (52.1)	93 (46.1)	259 (54.6)	**.040**	287 (53.4)	65 (47.1)	.190
> 3000	324 (47.9)	109 (53.9)	215 (45.4)		251 (46.6)	73 (52.9)	
Health Insurance (%)							
Yes	565 (83.6)	161 (79.7)	404 (85.2)	.076	450 (83.6)	115 (83.3)	.930
No/Unsure	111 (16.4)	41 (20.3)	70 (14.8)		88 (16.4)	23 (16.7)	
***Minority Stressors***							
Everyday Discrimination (SD)	2.06 (1.11)	2.10 (1.15)	2.05 (1.10)	.583	2.02 (1.03)	2.23 (1.39)	.056
Outness (%)							
Yes/ever coming out	549 (81.2)	156 (77.2)	393 (82.9)	.083	468 (87.0)	81 (58.7)	**<.001**
Never	127 (18.8)	46 (22.8)	81 (17.1)		70 (13.0)	57 (41.3)	
Rejection Anticipation (SD)	2.44 (1.02)	2.29 (0.95)	2.49 (1.04)	**.013**	2.44 (0.99)	2.38 (1.11)	.523
Identity Concealment (SD)	2.80 (1.09)	2.84 (1.07)	2.78 (1.09)	.527	2.77 (1.06)	2.91 (1.20)	.169
Internal Homophobia (SD)	1.63 (0.68)	1.77 (0.71)	1.66 (0.67)	.058	1.57 (0.61)	2.14 (0.77)	**<.001**
ACEs (SD)	1.00 (1.38)	1.12 (1.33)	0.96 (1.39)	.183	1.03 (1.42)	0.87 (1.20)	.253
Depressive symptoms (SD)	17.47 (10.59)	17.75 (10.74)	17.34 (10.54)	.643	17.56 (10.54)	17.09 (10.84)	.643
Social Support (SD)	5.07 (1.02)	4.99 (1.09)	5.11 (0.99)	.190	5.09 (1.01)	5.00 (1.09)	.361
Resilience (SD)	26.71 (8.45)	26.89 (8.45)	26.64 (8.38)	.724	26.77 (8.18)	26.49 (9.46)	.735

### Correlation statistics

The correlation matrix examining group differences between gays and bisexuals (Table 2) indicated that employment was the only significant correlate of cigarette smoking in both gay and bisexual subgroups. Among all psychosocial factors, group differences were only found regarding rejection anticipation and depressive symptoms. Thus, the two-group SEM included rejection anticipation, depressive symptoms, and cigarette smoking, predicted by employment (dummy coded as student or employed) (Fig. 2).

**Table 2:** Correlation matrix examining correlates of past 30-day cigarettes smoking among the full sample of Chinese sexual minority men, gays, and bisexuals, respectively (*N* = 676)

Variables	Total sample r coefficient, *p value* ***N*** = 676	Gay r coefficient, *p value* ***N*** = 538	Bisexual r coefficient, *p value* ***N*** = 138
***Sociodemographics***			
Age	0.063	0.061	−0.005
	*.102*	*.161*	*.949*
Place of Origin	0.034	0.033	0.008
	*.379*	*.448*	*.920*
Employment	0.185	0.157	0.240
	***<.001***	***<.001***	***0.004***
***Minority Stressors***			
Everyday Discrimination	0.021	0.030	−0.032
	.583	*.481*	*.710*
Outness	0.067	0.023	0.068
	*.084*	*.591*	*.423*
Rejection Anticipation	− 0.095	− 0.102	− 0.066
	***.013***	***.018***	*.444*
Identity Concealment	0.024	0.029	−0.015
	*.526*	*.497*	*.864*
Internal Homophobia	0.073	0.005	0.129
	*.058*	*.900*	*.129*
ACEs	0.054	0.054	0.082
	*.183*	*.232*	*.378*
Depressive symptoms	0.018	−0.032	0.199
	*.643*	*.459*	***.019***
Social support	−0.050	−0.046	− 0.051
	*.190*	*.291*	*.550*
Resilience	0.013	0.024	−0.013
	*.723*	*.575*	*.882*

### Sequential logistic regression models

Table 3 shows the results from four regression models. Model 1 result showed that compared to gay men, bisexual men were more likely to use cigarette (crude Odd Ratio [OR] = 1.76, 95% CI: 1.19–2.60). In model 2, bisexual men were still more likely to use cigarette compared to gay men (adjust OR = 1.79; 95% CI: 1.18–2.72). Compared to student, being unemployed was more likely to use cigarette (aOR = 3.96; 95% CI: 2.09–7.50). In model 3, only being unemployed (aOR: 3.69; 95% CI: 1.85–7.37) and rejection anticipation (aOR: 0.80; 95% CI: 0.65–1.00) were associated with cigarette use. In model 4, being unemployed was the only significant correlate of cigarette use (aOR: 3.78; 95% CI: 1.90–7.54). The majority of psychosocial factors were not associated with the outcome.

**Table 3:** Sequential logistic regressions identifying correlates of past 30-day cigarette use among gay and bisexual men in China (N = 676)

Variables	Model 1			Model 2			Model 3			Model 4		
	OR	CI	***p***	OR	CI	***p***	OR	CI	***p***	OR	CI	***p***
***Sexual Orientation***												
Gay (ref)												
Bisexual	1.76	1.19–2.60	**.004**	1.79	1.18–2.72	**.048**	1.50	0.92–2.45	.151	1.45	0.89–2.35	.138
***Sociodemographics***												
Age				1.00	0.98–1.03	.760	1.01	0.98–1.04	.676	0.99	0.97–1.02	.653
Rural/Urban												
Urban (ref)												
Rural				1.24	0.86–1.78	.250	1.26	0.85–1.87	.317	1.23	0.83–1.81	.310
Employment												
Student (ref)												
Employed				2.17	1.38–3.40	.655	1.98	1.22–3.22	.719	2.06	1.27–3.34	.779
Unemployed				3.96	2.09–7.50	**<.001**	3.69	1.85–7.37	**.001**	3.78	1.90–7.54	**.001**
***Psychosocial Risk Factors***												
Outness												
Yes (ref)												
Never							0.98	0.59–1.63	.950	0.97	0.58–1.61	.899
Depression							1.00	0.98–1.02	.642	1.00	0.98–1.02	.775
Everyday Discrimination							1.02	0.85–1.22	.844	1.03	0.85–1.25	.772
Rejection Anticipation							0.80	0.65–1.00	**.048**	0.81	0.65–1.01	.057
Internal Homophobia							1.17	0.85–1.60	.342	1.15	0.84–1.28	.387
Identity Concealment							1.03	0.84–1.27	.751	1.04	0.84–1.28	.744
ACEs							1.14	0.99–1.30	.058	113	0.98–1.30	.088
***Psychosocial Protective Factors***												
Resilience										1.01	0.98–1.04	.530
Social Support										0.97	0.78–1.20	.754

### Two-group SEM

#### Measurement model

We constructed unconstrained and constrained measurement CFA models. According to the model fit indices for the unconstrained model (χ2 = 1326.7 (576), *p* < .001; RMSEA = .061; CFI = .908; TLI = .896; SRMR = .056) and factor-loading constrained model (χ2 = 1358.2 (600), p < .001; RMSEA = .061; CFI = .907; TLI = .900; SRMR = .061), the chi-square difference test (χ2 = 31.5 [24]) was non-significant, indicating factor loading invariance. Therefore, we moved on to the structural analysis using this invariant measurement model.

#### Structural model

According to the model fit indices for the unconstrained model (χ2 = 971.0 (761), *p* < .001; RMSEA = .029; CFI = .920; TLI = .916; WRMR = 1.312) and constrained model (χ2 = 981.3 (770), *p* < .001; RMSEA = .028; CFI = .920; TLI = .916; WRMR = 1.397), the chi-square difference test (χ2 = 10.3 [9]) was non-significant, indicating structural invariance for gay and bisexual samples. The final two-group SEM fits the data well.

#### Final modeling results

**Fig. 3:**
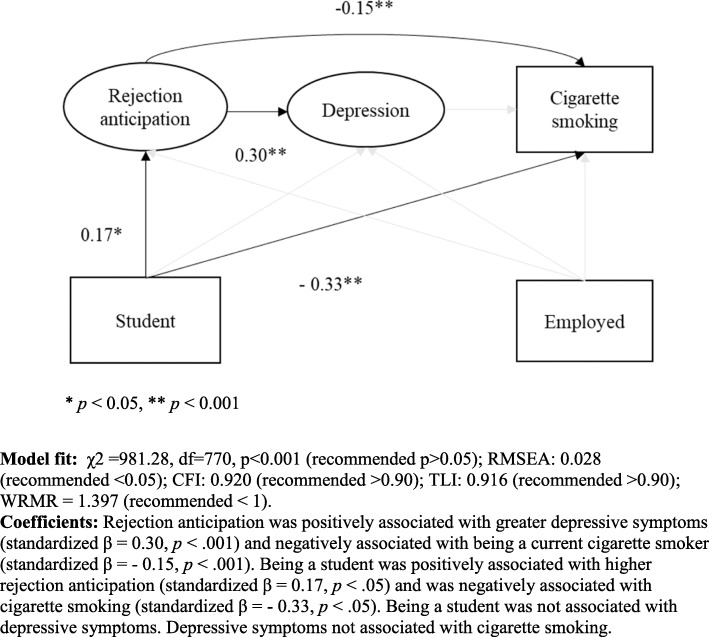
Final structural equation model testing pathway between rejection anticipation, depressive symptoms, and cigarette smoking among Chinese gay men and bisexual men. **Model fit:** χ2 = 981.28, df = 770, *p* < 0.001 (recommended *p* > 0.05); RMSEA: 0.028 (recommended < 0.05); CFI: 0.920 (recommended > 0.90); TLI: 0.916 (recommended > 0.90); WRMR = 1.397 (recommended < 1). **Coefficients:** Rejection anticipation was positively associated with greater depressive symptoms (standardized β = 0.30, *p* < .001) and negatively associated with being a current cigarette smoker (standardized β = − 0.15, *p* < .001). Being a student was positively associated with higher rejection anticipation (standardized β = 0.17, *p* < .05) and was negatively associated with cigarette smoking (standardized β = − 0.33, *p* < .05). Being a student was not associated with depressive symptoms. Depressive symptoms not associated with cigarette smoking

For both gay and bisexual men (Fig. 3), rejection anticipation was positively associated with greater depressive symptoms (standardized β = 0.30, *p* < .001) and negatively associated with being a current cigarette smoker (standardized β = − 0.15, *p* < .001). Being a student was positively associated with higher rejection anticipation (standardized β = 0.17, *p* < .05) and was negatively associated with cigarette smoking (standardized β = − 0.33, *p* < .05). Being a student was not associated with depressive symptoms. Depressive symptoms were not associated with cigarette smoking.

## Discussion

This study examined cigarette smoking among sexual minority men in China through the lens of the Minority Stress Theory. Our findings showed that bisexual men reported a higher rate of cigarette smoking compared to gay men (39.9% vs. 27.3%) in China. This is consistent with literature in other countries that bisexual men often report higher use of cigarette relative to gay men [79, 80].

Minority stress, particularly rejection anticipation, was positively associated with depressive symptoms in both gay and bisexual subgroups. This finding is consistent with studies suggesting that rejection anticipation is associated with the onset of depressive symptoms [81, 82]. In Chinese culture, being a majority represents righteousness and power, whereas minorities may be marginalized and/or ostracized [40]. Rejection anticipation was reversely associated with cigarette smoking. This unexpected result might be influenced by the launch of public smoke-free legislation in major cities like Beijing since 2014, that participants who anticipated rejections based on their sexual minority orientations might tend to minimize other types of rejections from the society including smoking. Taken together, rejection anticipation is a critical construct for sexual minority stress that is associated with important mental health outcomes, especially in a collective culture like China. We did not find associations between other minority stressors and depressive symptoms. However, a study in Thailand suggested that experiences of victimization, discrimination, and identify concealment also predicted depression among sexual minorities [83]. More research is needed to confirm the relationship between various minority stressors and depressive symptoms in different cultural contexts.

No direct relationship between depression and cigarette smoking among gay and bisexual men was indicated. However, other research has documented significant relationships between mental distress and smoking among sexual minority men globally [29, 84–86]. There might be some contextual factors – such as tobacco-free policy in our campus-based venues, peer influences, and social or cultural norms – confounding or buffering the effect of minority stressors on cigarette use in this study population. For example, structural discrimination was found to be a significant predictor of smoking among sexual minority subgroup [87]. Peer violence and pressure also could lead to tobacco use among sexual minority youth [88]. Future studies should consider the contextual influencing factors to better understand the effects of minority stressors on cigarette smoking.

We did not find distinctive inter-group differences between subgroups in the mechanism through which minority stress might affect cigarette smoking. According to a Chinese qualitative study on bisexuality, bisexual identity is a fairly new concept for many Chinese, especially among young adults and students, and bisexual individuals might undergo long time exploration of sexual identity and might experience enormous confusion and social pressure [89]. It may be that some young participants or student participants in our study might still be exploring their identities and assume themselves to be gay, bisexual, or heterosexual, depending on the sex of their current partners [56]. Thus, the distinction between gay men and bisexual men might be biased because of high proportion of students in our study. On the other hand, the staggering gender difference in smoking prevalence within the overall population in China (51.4% in men vs. 2.7% in women) may shed lights on the mechanism through which minority stress might affect smoking. In China, smoking behavior reflects the mainstream masculine norms and is more accepted and expected among men [90, 91]. Meanwhile, feminine devaluation is common across sexual minority communities [92]. Taken together, it is possible that Chinese bisexual men, who might have female sex partners, feel more pressured to adhere to masculine norms and are more likely to adopt masculine-associated behaviors such as smoking than gay men. Unfortunely, this study did not measure constructs related to gender norms or feminine/masculine identity. Thus, we could only speculate that gender norms or feminine/masculine identity may interact with internalized homophobia and contribute to the higher smoking rate in bisexual men. Further research is warranted to understand the extent to which the gender norms or feminine/masculine identity operates as an independent mediator or moderator on the pathway between internalized homophobia and smoking behaviors among Chinese sexual minority men.

Results indicated that being a student was positively associated with rejection anticipation among Chinese sexual minority men. Currently, no known study has evaluated the impact of school environments or policies on the health of sexual minority students in China, but empirical studies in the U.S. have found that sexual minority students experience higher rates of parental or peer rejections compared to heterosexual counterparts [93]. Moreover, 86.2% of U.S. sexual minority students experienced verbal harassment and 44.1% of these students experienced physical harassment [94]. In the U.S., sexual minority students felt less safe at school compared to heterosexual students [95]. Depending on the school climate, sexual minority students might be experiencing in a hostile or protective environment. Thus, more studies are needed to evaluate the impact of school setting toward their feeling of rejection, mental illness, and health risk behaviors among Chinese sexual minority students.

### Limitations

We used data from a convenient sample that was mainly comprised of urban, well-educated, and high-income gay/bisexual men, which might not be representative to all sexual minority men. Thus, our study provided little evidence base regarding the mental health status and smoking behaviors among LGBs residing in rural areas of China or who are with lower socioeconomic statuses. Also, the data were collected in sexual minority-serving organizations located in or close to college campuses, thus our measurement of cigarette smoking might be influenced by the smoke-free campus policies enforced since 2014 and therefore might not reflect the actual cigarette smoking prevalence. The cross-sectional nature of the data limits the inference regarding our findings, as SEM analysis using cross-sectional data might potentially misrepresent the pathway processes. Thus, researchers should use caution when interpreting our results. Future research should use longitudinal data to obtain better representation of the causal pathway between rejection anticipation and smoking among LGBs. Due to the page limitation of the survey instrument, we did not assess important factors that might influence smoking behaviors such as cultural aspects or peers or family influences. Gender norms and feminine/masculine identity were not considered in the study design. Thus, we couldn’t explore the roles of these constructs in influencing smoking behaviors among sexual minority men in China. Last, data were collected based on self-report using pen-and-paper and thus might be prone to recall bias and social desirability.

## Conclusions

Guided by Minority Stress Theory, this study is among the first to examine the minority stress – cigarette use pathway among a sample of Chinese gay and bisexual men. Our findings showed that bisexual men are more likely to smoke cigarette compared to gay men. Minority stress, particularly rejection anticipation, was associated with depressive symptoms in both gay and bisexual men, but depressive symptoms were not associated with cigarette smoking. Although we did not identify the pathway linking minority stress and cigarette smoking between gay and bisexual men, future studies can explore the role of minority stress and differences in cigarette smoking rates across sexual minority subgroups in urban and rural China and beyond in order to design tailored and targeted tobacco cessation interventions.

## Supplementary Information

**Additional file 1:.** China gay bisexual men study questionnaire__English. This file is the English version of the questionnaire used in this study (measurement only).

## Data Availability

The datasets used and/or analyzed during the current study are available from the corresponding author (Dr. Hong Yan) on reasonable request.

## Abbreviations

SEM: Structural equation modeling
SD: Standard deviation
OR: Odd Ratio
CI: Confidence Interval
CES-D: Center for Epidemiological Studies Depression Scale
ACEs: Adverse childhood experiences
ANOVA: Analysis of variance
CFA: Confirmatory factor analysis
SRMR: Standardized root mean square residual
CFI: Comparative fit index
TLI: Tucker–Lewis fit index
RMSEA: The root mean-square error of approximation
WRMR: Weighted root mean square residual
