# Supplementary material for: Minority stress, depression, and cigarette smoking among Chinese gay versus bisexual men: a two-group structural equation model analyses
Source: BMC Public Health. 2021 Jul 9;21:1358. doi: 10.1186/s12889-021-10888-5 (PMC8268265; doi:10.1186/s12889-021-10888-5)
Supplement: Supplementary file 1 — Additional file 1:. China gay bisexual men study questionnaire__English. This file is the English version of the questionnaire used in this study (measurement only). [file 12889_2021_10888_MOESM1_ESM.docx]

**0. demographics**

1. Age: ____ years old
2. Ethnicity:

A: Han B: Minorities:

1. Place of Origin:

A: Urban B: Rural

1. Education

A: Middle school or less B: senior high school C: undergraduate D: postgraduate

1. Occupation

A: Student B: Part-time Workers C: Full-time Workers D: Unemployed E: Other

1. Marital status

A: Not married B: Married C: Divorced

1. Income per month (RMB)

A: less than 500 B:500-1000 C:1001-3000 D:3001-6000 E: 6000 – 9000 F: > 9000

1. Do you have health insurance?

A: Yes B. No C. Unsure/unknown

1. Sexual orientation

A: Heterosexual B: Homosexual C: Bisexual D: Not sure

1. Have you ever “come out” to anyone?

A: Yes B. No C. Unsure/unknown

**1. Tobacco outcomes**

*Types of tobacco reference：*WHO’s Global Adult Tobacco Survey Chinese version; WHO’s International Tobacco Control Policy Evaluation Project China site

**
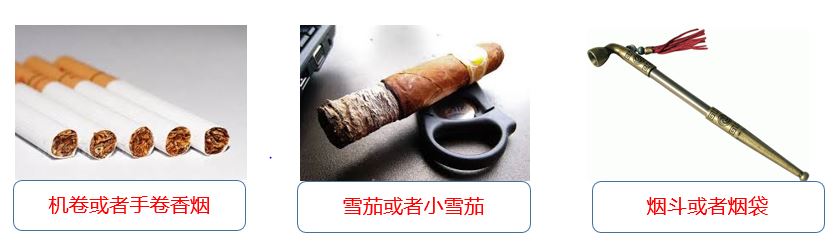
***Question format reference:*  DECOY survey

1) During the past 30 days, on how many days did you smoke cigarettes (even a puff)?

(please specify a number): ______days

2) During the past 30 days, on how many days did you smoke little cigars or cigarillos (even a puff)?

(please specify a number): ______days

3) During the past 30 days, on how many days did you smoke a pipe (even a puff)?

(please specify a number): ______days


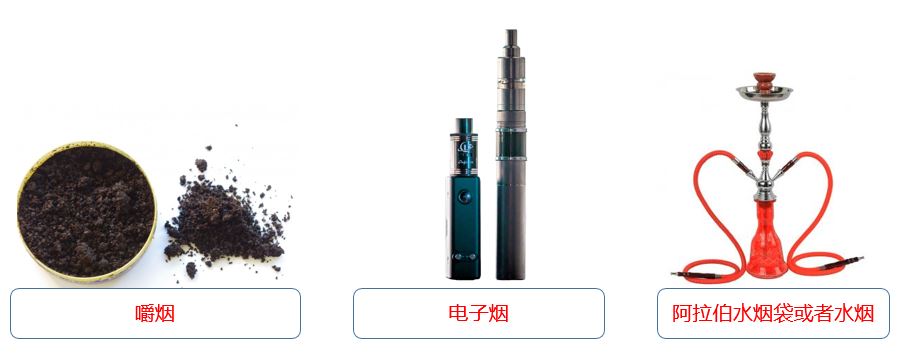


4) During the past 30 days, on how many days did you use chewing tobacco (even a bit)?

(please specify a number): ______days

5) During the past 30 days, on how many days did you smoke e-cigarette (even a puff)?

(please specify a number): ______days

6) During the past 30 days, on how many days did you use hookah (even a puff)?

(please specify a number): ______days

**2. Psychosocial scales**

**2.1 ACEs – 10 item**

|  |  | Yes | No | Refuse to answer |
| --- | --- | --- | --- | --- |
| 1 | Did a parent or other adult in the household often or very often… Swear at you, insult you, put you down, or humiliate you? or Act in a way that made you afraid that you might be physically hurt? |  |  |  |
| 2 | Did a parent or other adult in the household often or very often… Push, grab, slap, or throw something at you? or Ever hit you so hard that you had marks or were injured? |  |  |  |
| 3 | Did an adult or person at least 5 years older than you ever… Touch or fondle you or have you touch their body in a sexual way? or Attempt or actually have oral, anal, or vaginal intercourse with you? |  |  |  |
| 4 | Did you often or very often feel that … No one in your family loved you or thought you were important or special? or Your family didn’t look out for each other, feel close to each other, or support each other? |  |  |  |
| 5 | Did you often or very often feel that … You didn’t have enough to eat, had to wear dirty clothes, and had no one to protect you? or Your parents were too drunk or high to take care of you or take you to the doctor if you needed it? |  |  |  |
| 6 | Were your parents ever separated or divorced? |  |  |  |
| 7 | Was your mother or stepmother: Often or very often pushed, grabbed, slapped, or had something thrown at her? or Sometimes, often, or very often kicked, bitten, hit with a fist, or hit with something hard? or Ever repeatedly hit over at least a few minutes or threatened with a gun or knife? |  |  |  |
| 8 | Did you live with anyone who was a problem drinker or alcoholic, or who used street drugs? |  |  |  |
| 9 | Was a household member depressed or mentally ill, or did a household member attempt suicide? |  |  |  |
| 10 | Did a household member go to prison? |  |  |  |

**2.2 Perceived Social Support – 12 item**

|  |  | Very Strongly disagree | Strongly disagree | Mildly disagree | Neutral | Mildly agree | Strongly agree | Very Strongly agree |
| --- | --- | --- | --- | --- | --- | --- | --- | --- |
| 1 | There is a special person who is around when I am in need | 1 | 2 | 3 | 4 | 5 | 6 | 7 |
| 2 | There is a special person with whom I can share joys and sorrows | 1 | 2 | 3 | 4 | 5 | 6 | 7 |
| 3 | My family really tries to help me | 1 | 2 | 3 | 4 | 5 | 6 | 7 |
| 4 | I get the emotional help & support I need from my family | 1 | 2 | 3 | 4 | 5 | 6 | 7 |
| 5 | I have a special person who is a real source of comfort to me | 1 | 2 | 3 | 4 | 5 | 6 | 7 |
| 6 | My friends really try to help me | 1 | 2 | 3 | 4 | 5 | 6 | 7 |
| 7 | I can count on my friends when things go wrong. | 1 | 2 | 3 | 4 | 5 | 6 | 7 |
| 8 | I can talk about my problems with  my family | 1 | 2 | 3 | 4 | 5 | 6 | 7 |
| 9 | I have friends with whom I can share my joys and sorrows | 1 | 2 | 3 | 4 | 5 | 6 | 7 |
| 10 | There is a special person in my life who cares about my feelings | 1 | 2 | 3 | 4 | 5 | 6 | 7 |
| 11 | My family is willing to help me make decisions | 1 | 2 | 3 | 4 | 5 | 6 | 7 |
| 12 | I can talk about my problems with my friends | 1 | 2 | 3 | 4 | 5 | 6 | 7 |

| **2.3 Center for Epidemiologic Studies Depression Scale (CES-D 20)** | | | | | |
| --- | --- | --- | --- | --- | --- |
|  | **During the Past Week** | | | |  |
|  | Rarely or none of the time (less than 1 day ) | Some or a little of the time (1-2  days) | Occasionally or a moderate amount of time (3-4 days) | Most or all of the time (5-7 days) |  |
| 1. I was bothered by things that usually don’t bother me. | 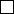 | 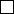 | 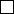 | 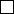 |  |
| 2. I did not feel like eating; my appetite was poor. | 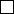 | 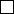 | 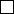 | 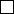 |  |
| 3. I felt that I could not shake off the blues even with help from my family or friends. | 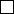 | 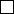 | 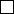 | 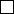 |  |
| 4. I felt I was just as good as other people. | 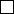 | 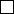 | 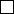 | 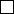 |  |
| 5. I had trouble keeping my mind on what I was doing. | 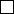 | 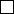 | 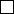 | 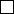 |  |
| 6. I felt depressed. | 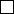 | 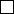 | 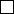 | 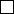 |  |
| 7. I felt that everything I did was an effort. | 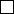 | 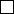 | 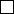 | 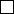 |  |
| 8. I felt hopeful about the future. | 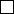 | 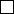 | 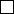 | 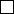 |  |
| 9. I thought my life had been a failure. | 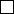 | 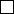 | 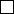 | 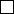 |  |
| 10. I felt fearful. | 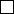 | 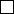 | 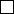 | 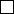 |  |
| 11. My sleep was restless. | 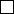 | 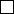 | 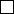 | 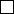 |  |
| 12. I was happy. | 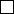 | 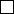 | 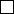 | 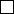 |  |
| 13. I talked less than usual. | 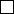 | 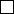 | 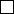 | 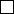 |  |
| 14. I felt lonely. | 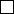 | 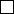 | 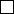 | 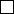 |  |
| 15. People were unfriendly. | 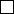 | 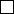 | 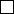 | 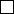 |  |
| 16. I enjoyed life. | 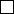 | 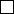 | 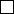 | 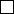 |  |
| 17. I had crying spells. | 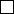 | 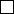 | 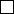 | 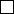 |  |
| 18. I felt sad. | 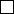 | 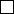 | 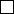 | 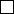 |  |
| 19. I felt that people dislike me. | 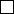 | 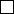 | 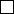 | 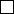 |  |
| 20. I could not get “going.” | 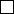 | 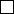 | 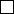 | 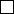 |  |
|  | | | | | |

**2.4 Everyday Discrimination Scale – 9 item**

Sometimes people feel as though they are treated differently than others. What are your experiences?

|  |  | Every day | Once a week | Several times a month | Several times a year | Less than yearly | Never |
| --- | --- | --- | --- | --- | --- | --- | --- |
| 1 | Are you treated with less courtesy than other people? | 1 | 2 | 3 | 4 | 5 | 6 |
| 2 | Are you treated with less respect than other people? | 1 | 2 | 3 | 4 | 5 | 6 |
| 3 | Do you receive poorer service in restaurants or stores? | 1 | 2 | 3 | 4 | 5 | 6 |
| 4 | Do people act as if they are better than you? | 1 | 2 | 3 | 4 | 5 | 6 |
| 5 | Do people act as if they are afraid of you? | 1 | 2 | 3 | 4 | 5 | 6 |
| 6 | Are you called names or insulted? | 1 | 2 | 3 | 4 | 5 | 6 |
| 7 | Are you threatened or harassed? | 1 | 2 | 3 | 4 | 5 | 6 |
| 8 | Do people act as if you are not smart? | 1 | 2 | 3 | 4 | 5 | 6 |
| 9 | Do people act as if you are dishonest? | 1 | 2 | 3 | 4 | 5 | 6 |

**2.5 Internalized Homophobia – 8 item**

How often do you have the following thoughts?

|  |  | Never | Sometimes | Often | Always |
| --- | --- | --- | --- | --- | --- |
| 1 | I often feel it best to avoid personal or social involvement with other gay/bisexual Men. | 1 | 2 | 3 | 4 |
| 2 | If someone offered me the chance to be completely heterosexual, I would accept the chance. | 1 | 2 | 3 | 4 |
| 3 | I wish I weren’t gay/bisexual. | 1 | 2 | 3 | 4 |
| 4 | I feel alienated from myself because of being gay/bisexual. | 1 | 2 | 3 | 4 |
| 5 | I wish that I could develop more erotic feelings about women. | 1 | 2 | 3 | 4 |
| 6 | I feel that being gay/bisexual is a personal shortcoming for me. | 1 | 2 | 3 | 4 |
| 7 | I would like to get professional help in order to change my sexual orientation from gay/bisexual to straight. | 1 | 2 | 3 | 4 |
| 8 | I have tried to become more sexually attracted to women. | 1 | 2 | 3 | 4 |

**2.6 Identity Concealment – 6 item**

Please indicate how frequently the following situation described occurs in your life.

|  |  | happens all of the time | happens a lot | happens sometimes | happens a little bit | never happens |
| --- | --- | --- | --- | --- | --- | --- |
| 1 | I avoid telling people about certain things in my life that might imply I am gay/bisexual. | 1 | 2 | 3 | 4 | 5 |
| 2 | I avoid talking about my romantic life because I do not want others to know I am gay/bisexual. | 1 | 2 | 3 | 4 | 5 |
| 3 | I change my mannerisms or speech because I do not want others to think I am gay/bisexual. | 1 | 2 | 3 | 4 | 5 |
| 4 | I do not bring a date to social events because I do not want others to know I am gay/bisexual. | 1 | 2 | 3 | 4 | 5 |
| 5 | I do not object when I hear anti- gay/bisexual remarks because I do not want others to assume I am gay/bisexual | 1 | 2 | 3 | 4 | 5 |
| 6 | I limit what I share on social media, or who can see it, because I do not want others to know I am gay/bisexual | 1 | 2 | 3 | 4 | 5 |

**2.7 Rejection Anticipation - 6 item**

Please indicate how frequently the following situation described occurs in your life.

|  |  | happens all of  the time | happens a lot | happens sometimes | happens a little bit | never happens |
| --- | --- | --- | --- | --- | --- | --- |
| 1 | When I meet someone new, I worry that they secretly do not like me because I am gay/bisexual | 1 | 2 | 3 | 4 | 5 |
| 2 | When I go out in public with my partner, I fear that people will treat us unkindly because I  am gay/bisexual | 1 | 2 | 3 | 4 | 5 |
| 3 | stay on guard and alert because something bad might happen to me because I am gay/bisexual | 1 | 2 | 3 | 4 | 5 |
| 4 | brace myself to be treated disrespectfully because I am gay/bisexual | 1 | 2 | 3 | 4 | 5 |
| 5 | I expect that others will not accept me because I am gay/bisexual | 1 | 2 | 3 | 4 | 5 |
| 6 | I worry about what will happen if people find out I am gay/bisexual | 1 | 2 | 3 | 4 | 5 |

**2.8 Connor-Davidson Resilience Scale (10-item CD-RISC)**

**Please rate the following statement based how you felt in the past month.**

|  |  | Not true at all | Rarely true | Sometimes true | Often true | True early all the time |
| --- | --- | --- | --- | --- | --- | --- |
| 1 | Able to adapt to change |  |  |  |  |  |
| 2 | Can deal with whatever comes |  |  |  |  |  |
| 3 | See the humorous side of things |  |  |  |  |  |
| 4 | Coping with stress strengthens |  |  |  |  |  |
| 5 | Tend to bounce back after illness or hardship |  |  |  |  |  |
| 6 | You can achieve your goals |  |  |  |  |  |
| 7 | Under pressure, focus and think clearly |  |  |  |  |  |
| 8 | Not easily discouraged by failure |  |  |  |  |  |
| 9 | Think of self as strong person |  |  |  |  |  |
| 10 | Can handle unpleasant feelings |  |  |  |  |  |
